# A Case of Post-COVID-19 Subacute Thyroiditis

**DOI:** 10.7759/cureus.12301

**Published:** 2020-12-26

**Authors:** Maham A Mehmood, Monica Bapna, Mahnoor Arshad

**Affiliations:** 1 Internal Medicine, Bronxcare Hospital, New York, USA; 2 Internal Medicine, Faisalabad Medical University, Faisalabad, PAK

**Keywords:** covid-19, thyroid disorder, thyroid-stimulating hormone, atenolol, prednisone

## Abstract

Subacute thyroiditis is usually a self-limiting inflammatory condition. The clinical presentation varies from person to person, but usually includes neck pain or discomfort and a painful diffuse goiter. There is at times a transient episode of hyperthyroidism followed by euthyroidism and sometimes hypothyroidism. We describe the case of a previously healthy 29-year-old female presenting with symptoms consistent with subacute thyroiditis. The patient had recently recovered from a mild episode of COVID-19 infection. Labs and imaging were consistent with the clinical diagnosis of subacute thyroiditis. The patient was provided symptomatic treatment with prednisone and atenolol and had an uneventful recovery.

## Introduction

The emergence of severe acute respiratory distress syndrome (SARS) in late 2019 and early 2020 posed a significant threat to the entire world, which started as a small cluster of cases in Wuhan, China, and subsequently spread across the globe in the form of a world pandemic. SARS-CoV-2 (COVID-19) was identified as the culprit. COVID-19 infection has a varied presentation from mild upper respiratory involvement to a more severe presentation, including acute respiratory distress syndrome, septic shock, and kidney failure. As of now, there have been a few cases that described the association between COVID-19 and thyroid gland involvement. We report a patient diagnosed with subacute thyroiditis after recovering from COVID-19 disease. The objective of the article is to create awareness regarding this novel entity and the association with thyroid dysfunction.

## Case presentation

A previously healthy 29 year old female presented to the ear, nose, and throat (ENT) clinic with fever and odynophagia. Her medical history was significant for coronavirus infection seven weeks prior, which was managed with a five-day course of azithromycin and hydroxychloroquine. She recovered without any complications. The patient was in her usual state of health ten days before the current presentation when she experienced pain with swallowing solid food, which gradually progressed to difficulty swallowing liquids and saliva. The fever was intermittent, associated with sweating with a maximum temperature of 102°F. She only took acetaminophen with minimal relief of symptoms. A review of the system was further positive for exertional tachycardia and shortness of breath, limiting her exercise tolerance to a block and unintentional weight loss of 10 lbs. The patient was a physician herself and remained at the frontlines in fighting the COVID-19 pandemic. She denied any history of smoking, alcohol, and illicit drug abuse. The family history was negative for any malignancy or thyroid disorder.

On index examination, the patient was febrile 101°F, tachycardiac at 130 beats/min, respiratory rate was 22/min, oxygen saturation was 98% on room air, and her blood pressure was normal. Her physical exam was notable for anterior neck tenderness, palpable left thyroid lobe compared to the right, and fine bilateral hand tremors. Her electrocardiography revealed sinus tachycardia. Endonasal endoscopy was negative, and the bedside ultrasound revealed a heterogeneously enlarged thyroid gland. Labs were notable for elevated erythrocyte sedimentation rate at 84 mm/hr, c-reactive protein at 44 mg/l, free thyroxine (T4) and total triiodothyronine (T3) levels were elevated at 4.4 ng/L (reference 0.6-1.3) and 374 ng/L (reference 80-150), respectively. Thyroid-stimulating hormone (TSH) was low at 0.01 mU/L. Thyroid peroxidase antibody and thyroid-stimulating immunoglobulin were negative. Her D dimer levels were <150 mg/l, COVID-19 PCR was negative, and the antibody was positive. Complete blood count and metabolic profile were normal. An endocrinologist was taken on board, a clinical diagnosis of subacute thyroiditis was made, and the patient was discharged home on oral prednisone (20 mg) and atenolol (25 mg) daily. The patient was followed at three days, and she remained symptomatic with exertional tachycardia and shortness of breath.

Echocardiography revealed normal ejection fraction and right ventricular systolic pressure, and her prednisone was increased to 40 mg and atenolol to 50 mg daily. The patient was followed at weekly intervals, with continued improvement. The prednisone was gradually tapered off over six weeks, and atenolol was discontinued. The patient remained asymptomatic at ten weeks follow up, and the thyroid function tests returned to normal.

## Discussion

Subacute thyroiditis most commonly affects females compared to males with a ratio of 4:1 and occurs at age range of 40-50 years [1,2]. The condition is recognized as self-limited inflammatory disorder of the thyroid gland that can manifest both during the active viral infection as well as with in two to eight weeks of recent viral infection [3]. The most commonly implicated viruses are coxsackievirus, mumps, measles, rubella, adenovirus, influenza, parvovirus B19, and many others [4]. The novel coronavirus has lately been recognized with growing awareness of the complications [5,6]. Brancatella et al. reported thyroiditis in an eighteen year old female after fifteen days of infection, symptoms resolved in a week and thyroid functions turned to baseline in forty days [5]. Thyrotoxicosis and COVID-19 infection share symptoms including sore throat, fatigue ,chills ,anorexia, fever and weight loss. This common symptomatology can be easily confused for COVID-19 symptoms [7]. Therefore, a strong clinical suspicion is required to rule out both diseases simultaneously. In a usual clinical course, half of the patients experience transient symptoms of thyrotoxicosis followed by euthyroidism, hypothyroidism, and normal thyroid function within three months [1,8].

In our literature research, in regards to the mechanism, we found that the inflammatory reaction to the virus activates the macrophages and cytotoxic T-cells, which then attack the viral damaged host tissues, followed by thyroid follicular cells, as they share the structural similarity [1]. Muller et al. suggest that the affinity of SARS-CoV-2 to the thyroid gland is via the angiotensin-converting enzyme 2 (ACE2) receptors. This receptor is recognized as necessary for SARS-CoV-2 to invade human cells and is more prevalent in thyroid cells than lung cells [9,10]. Hence, clinicians should be aware of the possibility of subacute thyroiditis in patients experiencing SARS-CoV-2 infection. Management of subacute thyroiditis is usually supportive and includes anti-inflammatory therapy with the nonsteroidal anti-inflammatory drug (NSAID) or prednisone. Symptomatic patients experiencing symptoms of hyperthyroidism such as palpitations, anxiety, or tremors may benefit from treatment with a beta-blocker such as propranolol/atenolol [11].

## Conclusions

We as physicians should keep the possibility of subacute thyroiditis in mind when assessing patients and not confuse thyroiditis symptoms for pharyngitis or lethargy that are usually present during COVID-19 infection, more importantly as most patients with SARS-CoV-2 are asymptomatic. It seems prudent to rule out SARS-CoV-2 infection in patients with symptoms suggesting thyroiditis, which would otherwise go unnoticed in the wake of this pandemic. 

## References

[1] Chong WH, Shkolnik B, Saha B, Beegle S (2020). Subacute thyroiditis in the setting of coronavirus disease 2019. Am J Med Sci.

[2] Bindra A, Braunstein GD (2006). Thyroiditis. Am Fam Physician.

[3] Fatourechi V, Aniszewski JP, Fatourechi GZ, Atkinson EJ, Jacobsen SJ (2003). Clinical features and outcome of subacute thyroiditis in an incidence cohort: Olmsted County, Minnesota, study. J Clin Endocrinol Metab.

[4] Desailloud R, Hober D (2009). Viruses and thyroiditis: an update. Virol J.

[5] Brancatella A, Ricci D, Viola N, Sgrò D, Santini F, Latrofa F (2020). Subacute thyroiditis after SARS-CoV-2 infection. J Clin Endocrinol Metab.

[6] Ippolito S, Dentali F, Tanda ML (2020). SARS-CoV-2: a potential trigger for subacute thyroiditis? Insights from a case report. J Endocrinol Invest.

[7] Guan W, Ni Z, Hu Y (2020). Clinical characteristics of coronavirus disease 2019 in China. N Engl J Med.

[8] Pearce EN, Farwell AP, Braverman LE (2003). Thyroiditis. N Engl J Med.

[9] Li MY, Li L, Zhang Y, Wang XS (2020). Expression of the SARS-CoV-2 cell receptor gene ACE2 in a wide variety of human tissues. Infect Dis Poverty.

[10] Muller I, Cannavaro D, Dazzi D (2020). SARS-CoV-2-related atypical thyroiditis. Lancet Diabetes Endocrinol.

[11] Campos-Barrera E, Alvarez-Cisneros T, Davalos-Fuentes M (2020). Subacute thyroiditis associated with COVID-19. Case Rep Endocrinol.

